# Science in stories: Implications for Latine children’s science learning through home-based language practices

**DOI:** 10.3389/fpsyg.2023.1096833

**Published:** 2023-02-23

**Authors:** Catherine A. Haden, Gigliana Melzi, Maureen A. Callanan

**Affiliations:** ^1^Department of Psychology, Loyola University Chicago, Chicago, IL, United States; ^2^Department of Applied Psychology, New York University, New York, NY, United States; ^3^Departmenst of Psychology, University of California, Santa Cruz, Santa Cruz, CA, United States

**Keywords:** storytelling, informal science learning, parent–child conversations, book reading, home learning, strengths-based

## Abstract

There is growing interest in stories as potentially powerful tools for science learning. In this mini-review article, we discuss theory and evidence indicating that, especially for young children, listening to and sharing stories with adult caregivers at home can make scientific ideas and inquiry practices meaningful and accessible. We review recent research offering evidence that stories presented in books can advance children’s science learning. Nonetheless, most of this work focuses on middle-class European-American U. S. children and involves narrative story books. Given the national imperative to increase Latine^1^ representation in STEM education and career pursuits in the U. S., we argue that it is vital that we broaden the definition of stories to include oral narrative storytelling and other conversational routines that Latine families engage in at home. Cultural communities with firmly rooted oral traditions, such as those from Latin American heritage, rely frequently on oral storytelling rather than book reading to convey world and community knowledge to young children. Therefore, we advocate for a strengths-based approach that considers Latine families’ everyday practices around science and storytelling on their own terms instead of contrasting them with European-American middle-class practices. We offer support for the view that for young children in Latine communities, culturally relevant oral practices, including personal narrative storytelling, can engender significant opportunities for family science learning at home.

## 1. Introduction

In this mini-review article, we focus on the ways stories can advance children’s science learning opportunities at home. Stories are culturally determined ways of communicating lived or imagined experiences (Bruner, 1996). Whereas most research and educational practice regarding stories centers on fostering language and literacy skills, there is growing interest in and evidence for stories supporting informal and formal science education (e.g., Brophy et al., 2008; Avraamidou and Osborne, 2009; Dahlstrom, 2014; Wilson-Lopez and Gregory, 2015; Cunningham, 2018). As we discuss, theory and evidence indicates that especially for young children, sharing stories can be a powerful vehicle for informal science learning in families. Paralleling the language and literacy work, most of the research on stories for science learning centers on story book reading. From our perspective, focusing on stories in books is not sufficient to realize the potential of stories to offer accessible and equitable science learning opportunities for young children. We argue it is necessary to broaden the focus on stories to include oral storytelling and other conversational routines that families engage in at home. This is especially important when we consider that among cultural communities with firmly rooted oral traditions, including those from Latin American heritage, oral storytelling rather than book reading may be a more common everyday practice for conveying knowledge to young children (Billings, 2009; Sánchez, 2009; Reese, 2012; Melzi et al., 2019). Unfortunately, the overreliance on print communication in formal and informal learning contexts often excludes such culturally relevant oral-based practices that can support children’s science learning. What is needed is an emphasis on *ciencia en relatos* - science in stories - that includes understanding and leveraging oral practices that are cultural resources for supporting Latine children’s science learning at home.

The motivation for this review is three-fold. First is the national imperative to broaden participation in science, technology, engineering, and mathematics (STEM). The U.S. Census Bureau reports that the Latine population reached 64.1 million in 2020 and is estimated to increase to 111 million by 2060, or nearly 28% of all Americans (Vespa et al., 2020). As the U.S. Latine population is increasing at a rapid pace, so too is the percentage of Latine students attending and graduating from college (Hussar et al., 2020). However, whereas 56% of the bachelor’s degrees in science fields go to White Non-Latine students, only 13.5% are awarded to Latine students (National Science Foundation, 2019). To broaden participation in STEM education of groups underrepresented in STEM, we need to identify and promote strategies that respond to and value the experiences and funds of knowledge that students bring from their homes and communities (González et al., 2013; Bricker and Bell, 2014; Hernández et al., 2016). In particular, we know little about the socio-cultural and familial experiences of Latine children that can contribute to their early science skills and learning.

Second, research has established that early informal learning experiences in homes, museums, and libraries can foster lasting interest and knowledge of STEM (e.g., National Research Council [NRC], 2009, 2012; Sobel and Jipson, 2016; National Academies of Sciences, Engineering, and Medicine [NAS], 2018). For instance, parents’ support of children’s engagement with science activities predicts children’s developing attitudes and later participation in science (Alexander et al., 2012). Further, parents’ elaborative talk about science topics is related to children’s engagement during hands-on activities, their later learning and memory of science-related experiences, and their interest in science (Tenenbaum et al., 2005; Benjamin et al., 2010; Jant et al., 2014; Callanan et al., 2017). Science practices such as asking questions, observing, explaining, and making predictions are both strengths of young children’s everyday curiosity (Callanan and Oakes, 1992) and building blocks for more advanced STEM thinking (NGSS Lead States, 2013). Essentially, STEM learning opportunities involving family interactions at home and in other informal educational settings can open doors to future STEM educational and career paths. Nonetheless, such benefits may not be realized without uncovering and building on the experiences and practices of culturally and linguistically diverse families.

Third, although there is growing attention to science learning in Latine populations, too often this work takes a deficit approach by comparing Latine children with white, middle-class children and focusing on “gaps” in knowledge or practices (e.g., fewer books, less book reading). Instead, we advocate for work that can contribute to the strengths-based literature (e.g., Gutiérrez and Rogoff, 2003; Bang et al., 2012; Solis and Callanan, 2016, 2018), considering Latine families’ everyday practices around science and stories on their own terms. For Latine families in the U.S., sharing oral stories is pervasive in everyday routines, and firmly rooted in Latin American oral traditions (McDowell et al., 1993; Delgado-Gaitan, 1994; Sánchez, 2009). Moreover, consistent with sociocultural perspectives on development (e.g., Vygotsky, 1978; Rogoff, 1990), the social-linguistic milieu of shared reading, storytelling, and other conversational routines can provide a setting for children’s learning. This perspective drives a focus on culturally relevant oral practices, including personal storytelling, and efforts to understand how these social interactions can engender authentic and meaningful opportunities for Latine families’ science learning at home.

Given the applied significance of our topic, the review that follows illustrates the ways that stories in books *and* those told orally by families at home can provide rich opportunities for science learning, and underscores the implications for broadening STEM participation among Latine children.

## 2. Stories for science learning

Notwithstanding the research and educational practices centering on stories for promoting language and literacy skills (e.g., Reese, 1995; Sénéchal, 2015; Wasik et al., 2016; Flack et al., 2018), there is a growing need to identify whether and how stories can support other academic skills, especially science learning. As with the work on language and literacy, the current research on stories for science learning focuses mostly on books, and involves white, middle-class U.S. children. However, we must build on Bruner’s (1991) idea that oral storytelling is a natural form of human understanding that perhaps is more engaging for children and adults than scientific prose. Doing so encourages serious consideration of everyday home-based language practices of Latine families for whom stories in books may not be a primary way of conveying knowledge. Importantly, stories in books *and* told orally can convey science information that might not be available through direct experience and can boost children’s engagement with challenging science-related ideas (Kelemen et al., 2014; Browning and Hohenstein, 2015; Evans et al., 2016; Cho and Plummer, 2018). Stories also can be especially potent for making scientific ideas and inquiry practices meaningful and accessible (Graesser et al., 1980; Avraamidou and Osborne, 2009; Frykman, 2009; Klassen, 2010). By helping children connect with and see the importance of science problems, and how general and abstract science concepts can be applied to situations that are relevant to them, stories can motivate interest in and learning of science (Cordova and Lepper, 1996; Willingham, 2009; Murmann and Avraamidou, 2016) Moreover, stories can provide a springboard for elaborative discussions of science topics, involving cognitively challenging utterances about science and ideas, and scaffolding engagement in practices of science by caregivers and children (Haden, 2010; Solis and Callanan, 2018; Plummer and Cho, 2020; Shirefley et al., 2020). Embedding science information in stories can make representations of science-related knowledge and experiences stronger, more concrete, and meaningful (Haden et al., 2016; Marcus et al., 2023, in review). In these and other myriad ways, shared book reading and oral storytelling offer powerful mechanisms for children’s science learning at home.

### 2.1. Science in books

Most of the work on science learning through book reading has involved empirical studies in which researchers read story books to children to teach biological (e.g., Tare et al., 2010; Ganea et al., 2011, 2014; Waxman et al., 2014; Walker et al., 2015; Strouse and Ganea, 2021) and physical science information (Venkadasalam and Ganea, 2018; Ganea et al., 2021). For example, Kelemen et al. (2014) found gains in 5- to 8-year-olds’ understanding of natural selection after reading a storybook that conveyed the concept in narrative form. This was reflected not only in more accurate answers to test questions, but also more logical and coherent explanations applied to novel species, as well as children’s retention of their increased understanding over 3 months. Other work shows that despite concerns that fictional story books could interfere with children’s learning of science content (Ganea et al., 2014; Walker et al., 2015), fantastical content in story books might not hinder, and may even improve young children’s participation and engagement with science-related ideas (Hopkins and Lillard, 2021; Hopkins and Weisberg, 2021; Richert and Schlesinger, 2022). Some scholars have proposed that narrative story books may be a more engaging and productive way to communicate science topics and scientific processes to learners than typical scientific expository texts (Kurth et al., 2002; Avraamidou and Osborne, 2009; Glaser et al., 2009; Dahlstrom, 2014).

Caregivers report that they primarily share narrative story books at home (Price et al., 2009; Robertson and Reese, 2017), although children may not have strong preferences for one or the other book type (Kotaman and Tekin, 2017). Theory and research in early education emphasizes offering young children a “balanced diet” of narrative, expository and other types of texts to support learning (Teale, 2003; Pentimonti et al., 2010; Robertson and Reese, 2017). Nonetheless, the use of expository texts with young children to relay factual information is increasing in educational settings (Saracho, 2017; Bergman Deitcher et al., 2019). Moreover, research shows that caregivers use more cognitively demanding questions, emphasize new vocabulary, and their children talk more, during shared reading interactions involving expository as compared to narrative books (e.g., Pellegrini et al., 1990; Price et al., 2009; Zucker et al., 2010). Some researchers and educators suggest that expository texts may be especially supportive of lasting learning, enabling the transfer of science information conveyed in books to present and future learning opportunities (Ganea et al., 2008, 2011; Richert and Smith, 2011; Kotaman and Tekin, 2017).

Although direct comparisons of science learning from narrative and expository texts are rare, some studies favor one or the other genre, whereas other studies indicate comparable or complementary science learning from both types of books (Torr and Clugston, 1999; Gonzalez et al., 2010; Pollard-Durodola et al., 2015; Nevo and Vaknin-Nusbaum, 2018). To illustrate the mixed results, consider that in Browning and Hohenstein (2015), 5- to 7-year-olds who were introduced to evolution using a narrative text expressed deeper understanding than did those introduced to the same ideas through expository text. In contrast, Walker et al.’s (2015) preschool-aged participants were more likely to generalize causal biological information from picture books to real world situations when they had learned the information from a realistic compared to a fantasy story context. For the 4 to 5-year-olds in Venkadasalam and Ganea (2018), genre did not predict science learning, so long as the books were similarly engaging and provided accurate information. Likewise, Aydin et al. (2021) tested 3- to 5-year-old children’s learning of factual information about animals based on hearing both a storybook that contained anthropomorphism and a book that was non-narrative and did not include fantastical elements. Preschoolers in this study learned new facts about animals from both types of books. However, when the information in the narrative and expository books conflicted, older preschoolers tended to report information from the expository text; younger preschoolers showed no prioritization of information learned from one or the other book type.

### 2.2. Science stories and hands-on learning

A primary way that young children engage in science learning is through direct experience interacting with objects and the natural world (e.g., Piaget, 1970; Marin and Bang, 2018), and this is reflected in many early science educational opportunities for children in and out of school. When stories are combined with hands-on activities, stories can provide mechanisms for learning beyond what children might gain from hands-on engagement alone. To illustrate, several early childhood curricula pair book reading and hands-on STEM activities. Some involved specially crafted STEM-focused story books that provide visual depictions of math or engineering ideas, present problems for children to explore, and feature models for math or engineering investigations (Casey et al., 2004; Cunningham, 2018; Svarovsky et al., 2018). Engineering is Elementary curriculum units (www.eie.org; Cunningham and Lachapelle, 2014) begin with story books set in countries around the world in which the elementary-school protagonists solve problems with the help of adult engineers. There is evidence that these programs are effective, and in some cases, girls and children from groups underrepresented in STEM show particularly high learning gains (Cunningham, 2018; Svarovsky et al., 2018).

There are also an increasing number of researchers and educators seeking to understand the ways that stories in books, oral narratives, or picture-based narrative formats can advance informal STEM learning opportunities for children at home, and in libraries and museums (Pattison et al., 2020). In several studies, combining book reading or oral narratives with hands-on STEM activities in informal settings supported children’s increased interest and knowledge of STEM (e.g., Luke et al., 2010; Evans et al., 2016; Murmann and Avraamidou, 2016; Pattison et al., 2017, 2018; Tzou et al., 2019; Plummer and Cho, 2020; Letourneau et al., 2022). As another example, in Callanan et al. (2021), some families engaged with a hands-on story-based museum exhibit that conveyed a non-verbal narrative about the life and death of a mammoth. These families, in turn, talked more about science in related exhibits containing fossilized mammoth bones, than those who did not use the story-based exhibit. In other work, oral stories told by STEM experts fostered family STEM learning conversations during hands-on museum and library programs (Siegel, 2019; Zimmerman et al., 2018; Solis et al., 2023, in preparation). Notably, although these latter studies connecting stories and hands-on activities have primarily focused on white, middle-class families, they do support a move to transcend book reading to understand the ways that oral stories can provide science learning opportunities for children.

## 3. Science in stories: Implications for Latine children

As this brief review indicates, science books can be used to support children’s science learning. But there is still much to learn about the ways caregivers and children engage in science talk while reading science-related narrative and expository texts. Extratextual talk that goes beyond the printed word is likely important for science learning, just as it has been linked to development of specific oral language and early literacy skills (Haden et al., 1996; Fletcher and Reese, 2005; Hindman et al., 2008; Mol et al., 2008; Zucker et al., 2013). However, it is also the case that the few available studies with Latine families suggest that there may be distinctive patterns of associations between parental language during book sharing and child language outcomes with these families (e.g., Caspe, 2009; Escobar et al., 2017; Schick et al., 2017; Melzi et al., 2019). In these studies, Latine parents generally use less extratextual talk and fewer questions while sharing books with their children.

We need to address the serious gaps in current knowledge about the ways that caregivers from culturally and linguistically diverse backgrounds, and particularly Latine communities, may engage with science as they read books with their children. Nevertheless, this step is not enough if we want to capitalize on Latine family practices as points of leverage to support children’s understanding of and interest in science. By broadening our consideration of science in stories to capture oral storytelling and other conversational routines, it is possible to gain purchase on the ways stories are cultural resources for Latine children’s science learning at home.

While we acknowledge the diversity of Latine families as a result of their immediate and broader ecologies (e.g., country of origin, immigration histories, rural vs. urban upbringing, languages spoken), we also believe that Latine families share a set of core values and lived experiences, among these the widespread preference for oral practices. Ethnographic work in U.S. Latine communities, for example, shows that adult family members frequently use oral stories to impart lessons about life, provide education related to moral and social issues, and transmit cultural beliefs, values, and attitudes to their children (Delgado-Gaitan, 1994; Delgado-Gaitán, 2004; Espinoza-Herald, 2007; Cortez, 2008; Sánchez et al., 2010; Solis, 2017). Family reminiscing (i.e., conversations about shared past events), traditional stories marked by *dichos* (i.e., popular sayings), as well as *consejos* (advice), *refranes* (proverbs), and *adivinanzas* (riddles) are forms of oral discourse that Latine families use to support children’s learning (Melzi et al., 2019). Work in Latin American communities outside of the U.S. documents a similar preference in families of young children. For instance, in Melzi and Caspe (2005) Spanish-speaking urban Peruvian families reported inventing and telling oral stories to their preschoolers more frequently than did English-speaking urban U.S. European-American families, who preferred book sharing. These everyday oral practices are formative, with research showing that oral sharing of stories with preschoolers predicts children’s school readiness, including oral language and early literacy skills (Reese, 1995; Melzi et al., 2022) and cognitive abilities (Fivush et al., 2006). Yet, all too often, oral practices of culturally and linguistically diverse families are overlooked.

A focus on STEM-related oral practices among Latine families is supported by a growing body of research. Consistent with other work involving families of diverse educational and economic backgrounds (Bang and Medin, 2010; Solis and Callanan, 2016, 2021; Calabrese Barton and Tan, 2020; Huitzilopochtli et al., 2021), Latine families often engage in conversations about nature, and especially animals, plants, weather, and astronomy (Pérez-Granados and Callanan, 1997; Kelemen et al., 2005; Callanan et al., 2019; Shirefley et al., 2020; Castañeda et al., 2022). *Adivinanzas* (riddles) are used to entertain children, but they can rely on nature and other science-related topics thereby fostering children’s knowledge and engaging them in science practices (e.g., analysis, explanation, interpretation). For example, in the following *adivinanza* (from Arreguín-Anderson and Ruiz-Escalante, 2018) the idea that plants have basic needs is conveyed through a simple riddle:

**Table 1:** 

Adivinanza	Riddle
Siempre mirando al sol Y no soy un caracol. Giro y giro sin fin Y no soy un bailarín.	I always turn to the sun But I am not a snail, I endlessly turn, But I am not a dancer.
Respuesta: El girasol	Answer: The sunflower

Similarly, *dichos* (sayings) are told in families’ homes to transmit wisdom and moral education. Some of these *dichos* are inspired by nature and people’s interactions with nature. Thus, they provide opportunities for adults to explain the nature analogies to children, and in doing so expand their knowledge about life and science (Arreguín-Anderson and Ruiz-Escalante, 2018).

Looking ahead, we must advance current understanding of stories as cultural resources for Latine families’ science learning at home. Doing so will not be easy because it requires moving away from a focus on book reading as a primary source of stories, as well as developing clearer understandings of how stories connect with hands-on activities in children’s lives. Those of us who study stories for science learning need not to repeat mistakes of research concerning shared book reading and early literacy skills that sought to change Latine caregivers’ behaviors, in turn, failing to produce the desired outcomes (see Melzi et al., 2019, for review). Efforts to support children’s science learning are more likely to be successful when they build upon families’ practices rather than seeking to replace them (cf. Melzi et al., 2022). However, insights into science in stories might still be limited without a corresponding expansion - even a “desettling” (Bang et al., 2012) - of definitions of what counts as science (see Huitzilopochtli et al., 2021; Pattison et al., 2022, for similar arguments). If we take a strengths-based approach that values the ways that science is manifested in Latine families’ stories, it should be possible to uncover the science in stories that are part of these children’s everyday, home-based language practices. Doing so will enable us to leverage their unique experiences to support Latine children’s science learning, and ultimately, broaden Latine children’s participation in STEM.

## Author contributions

All authors developed the structure and content of the manuscript. All authors contributed to the article and approved the submitted version.

## Funding

This research was supported by the National Science Foundation (NSF) under a collaborative grant DRL #2055382 (PI: GM), # 2055345 (PI: CH), and # 2055426 (PI: MC).

## Conflict of interest

The authors declare that the research was conducted in the absence of any commercial or financial relationships that could be construed as a potential conflict of interest.

## Publisher’s note

All claims expressed in this article are solely those of the authors and do not necessarily represent those of their affiliated organizations, or those of the publisher, the editors and the reviewers. Any product that may be evaluated in this article, or claim that may be made by its manufacturer, is not guaranteed or endorsed by the publisher.
